# Control of intrinsic pacemaker frequency and velocity of colonic migrating motor complexes in mouse

**DOI:** 10.3389/fnins.2014.00096

**Published:** 2014-05-08

**Authors:** Kyra J. Barnes, Elizabeth A. Beckett, Simon J. Brookes, Tiong Cheng Sia, Nick J. Spencer

**Affiliations:** ^1^Discipline of Human Physiology, Center for Neuroscience, School of Medicine, Flinders University of South AustraliaAdelaide, SA, Australia; ^2^Discipline of Physiology, School of Medical Sciences, University of AdelaideAdelaide, SA, Australia

**Keywords:** colon, enteric nervous system, peristalsis, migrating motor complex, myenteric plexus

## Abstract

The mechanisms that control the frequency and propagation velocity of colonic migrating motor complexes (CMMCs) in mammals are poorly understood. Previous *in vitro* studies on whole mouse colon have shown that CMMCs occur frequently (~every 1–3 min) when the colon is devoid of all fecal content. Consequently, these studies have concluded that the generation of CMMCs and the frequency which they occur does not require the presence of fecal content in the lumen. However, in these studies, stimuli have always been unavoidably applied to these empty colonic preparations, facilitating recordings of CMMC activity. We tested whether CMMCs still occur in empty whole colonic preparations, but when conventional recording methods are not used. To test this, we used video imaging, but did not utilize standard recording methods. In whole isolated colons containing multiple endogenous fecal pellets, CMMCs occurred frequently (1.9 ± 0.1/min) and propagated at 2.2 ± 0.2 mm/s. Surprisingly, when these preparations had expelled all content, CMMCs were absent in 11/24 preparations. In the remaining preparations, CMMCs occurred rarely (0.18 ± 0.02/min) and at reduced velocities (0.71 ± 0.1 mm/s), with reduced extent of propagation. When conventional recording techniques were then applied to these empty preparations, CMMC frequency significantly increased, as did the extent of propagation and velocity. We show that in contrast to popular belief, CMMCs either do not occur when the colon is free of luminal contents, or, they occur at significantly lower frequencies. We believe that previous *in vitro* studies on empty segments of whole mouse colon have consistently demonstrated CMMCs at high frequencies because conventional recording techniques stimulate the colon. This study shows that CMMCs are normally absent, or infrequent in an empty colon, but their frequency increases substantially when fecal content is present, or, if *in vitro* techniques are used that stimulate the intestine.

## Introduction

Colonic migrating motor complexes (CMMCs) are cyclical contractions of the colonic smooth muscle that propagate over significant distances of large intestine; are dependent upon the enteric nervous system; and are thought to aid in the propulsion of colonic contents. CMMCs have been reported to occur in a variety of mammals, including the human colon both *in vivo* (Hagger et al., 2002) and *in vitro* (Spencer et al., 2012). They also occur reliably in the isolated colon of rat (Ferre and Ruckebusch, 1985) and mouse (Wood, 1973; Brann and Wood, 1976; Fida et al., 1997; Bush et al., 2000, 2001; Brierley et al., 2001; Roberts et al., 2007; Copel et al., 2013; Spencer et al., 2013). Over the past decade, the mouse colon has proved an ideal model species for recording CMMC activity, at least *in vitro*, because CMMCs occur consistently and rhythmically in the isolated whole colon (Fida et al., 1997; Bush et al., 2000; Brierley et al., 2001; Roberts et al., 2007; Spencer et al., 2013). In isolated whole mouse colon, CMMCs occur every 1–3 min (Fida et al., 1997; Brierley et al., 2001; Bush et al., 2001; Roberts et al., 2008; Spencer et al., 2013). Although the pacemaker cell(s) responsible for CMMC generation have not been identified, it is clear that the pattern generator underlying CMMC generation and frequency must lie within the myenteric plexus and/or muscularis, since removal of the mucosa and submucosal plexus does not block their generation (Keating and Spencer, 2010; Zagorodnyuk and Spencer, 2011; Spencer et al., 2013). At present, the functional role of CMMCs remains unclear, but it is likely they provide a propulsive force facilitating movement of intraluminal content.

To date, *in vitro* mechanical recordings of mouse CMMC activity have been made from empty segments of whole colon, where the luminal contents have been flushed free (Bush et al., 2000; Brierley et al., 2001; Powell and Bywater, 2001; Roberts et al., 2007; Spencer et al., 2013). It has been presumed that because CMMCs occur reliably and frequently from these empty preparations, that CMMC generation is determined independently of the presence of fecal contents. However, in all these previous studies, some form of external stimulus has always been applied to the gut wall, in order for mechanical recordings of CMMC activity to be made (Fida et al., 1997; Brierley et al., 2001; Bush et al., 2001; Roberts et al., 2008; Spencer et al., 2013). We have suspected that these mechanical recordings could themselves have induced CMMCs in the absence of luminal contents, giving the false impression that CMMCs occur frequently in empty segment of whole colon. The types of *in vitro* stimulation that have been applied to the colon to facilitate mechanical recordings in the past have been in the form of metal spring clips (used to pinch the bowel) see (e.g., Fida et al., 1997; Bush et al., 2000; Spencer et al., 2013), or simply by distending the oral and anal cut ends of the colon, caused by mounting the preparation into and organ bath (Bush et al., 2000; Brierley et al., 2001; Powell and Bywater, 2001; Spencer et al., 2013). Also, all previous mechanical recordings have been made with a degree of resting stretch or tension applied to the colonic wall. This made us speculate that these stimuli may have inadvertently induced, or modified CMMC characteristics in empty segments of large bowel.

The major aim of this study therefore was to determine whether the characteristics of CMMCs reported in previous studies are modified, or induced, by the recording apparatus that has been traditionally used to detect this motor pattern *in vitro*. To address this question, we have placed the entire isolated mouse colon into an organ bath, but avoided using a conventional organ bath that stimulates the oral and anal ends of the preparation. This is also without using any conventional mechanical clips that pinch the bowel, enabling mechanical recordings to be made. Instead of these methods, we have used video imaging of colonic wall movements, which does not require any contact with the bowel itself. We report the unexpected observation that CMMCs occur rarely, or not at all, in isolated whole segments of mouse colon that lack luminal contents; and when conventional recording methods are not utilized.

## Methods

### Preparation of tissues

Adult mice between 30 and 120 days of age were euthanized by inhalation overdose of isofluorane followed by exsanguination, in a manner approved by the Animal Welfare Committee of Flinders University. The entire colon was removed and placed in a petri dish filled with warm (25–30°C) Krebs solution constantly bubbled with carbogen gas (95% O_2_/5% CO_2_). The mesentery was carefully trimmed free and the entire colon, containing natural pellets, was placed in an organ bath at 35.5 ± 0.5°C. The whole colon was anchored with a single stainless steel pin (<700 μm diameter) at the oral and anal ends which did not interfere with movement of pellets in the lumen. The time taken for removal of colon from animal to the time at which the colon was anchored in the organ bath was typically <5 min.

### Video recordings made from empty and stimulated segments of mouse colon

The typical duration of video recordings of expulsion of faecal pellets was up to 30 min. After expulsion of natural pellets had been recorded, the colon was gently flushed with 5 ml warm Krebs, and left to equilibrate for 30 min. This procedure was necessary since not all colon preparations had fully expelled faecal pellets within the 30 min of video recordings. To maintain consistency for comparison, preparations that did fully empty were also gently flushed with warm Krebs.

**Figure 1:**
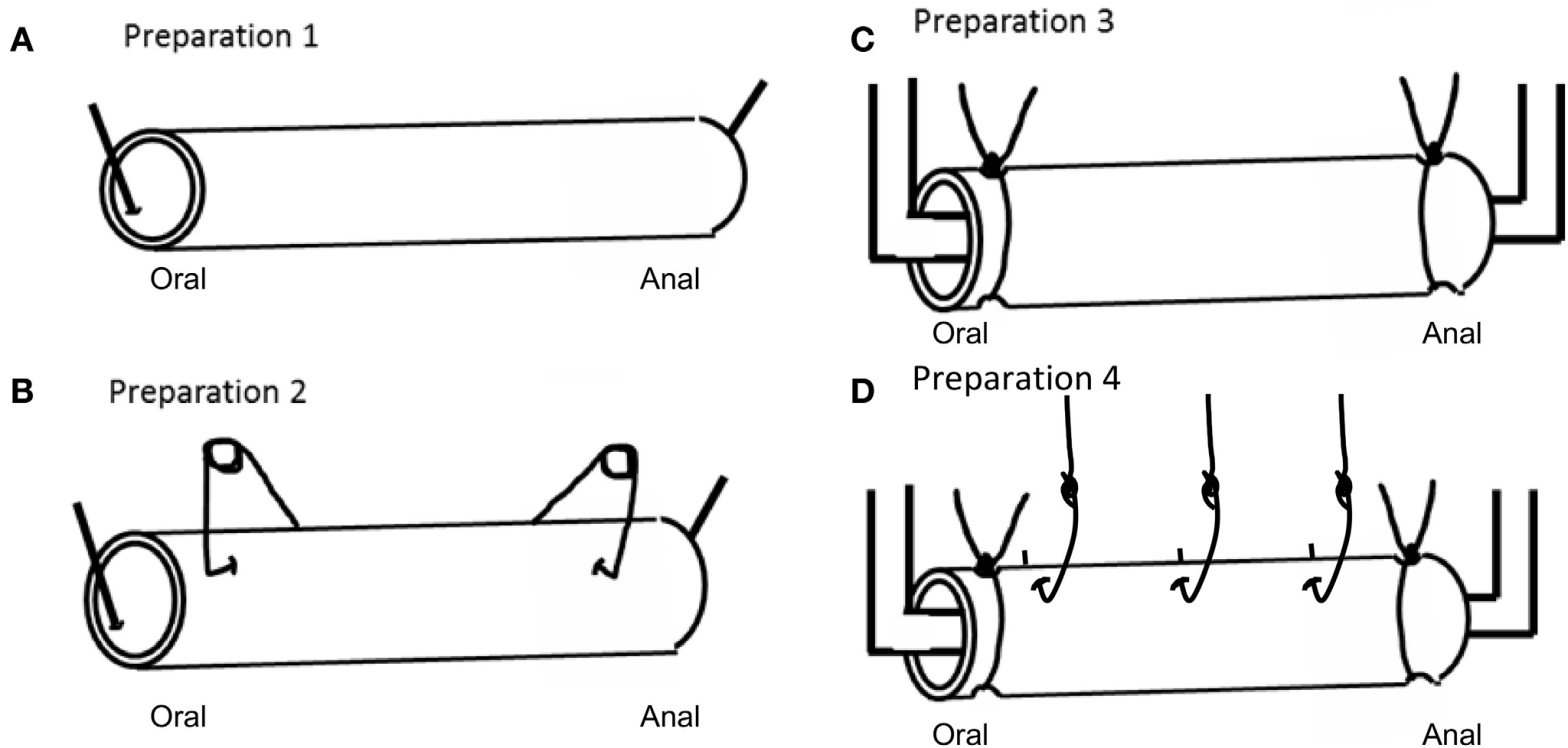
**Different types of preparations used to record CMMC activity in isolated whole segments of mouse colon. (A)** Preparation 1 is a novel preparation, where video recordings are made from the empty whole colon, with only a single pin inserted at proximal and distal end to anchor the preparation to the colon. No force transducer clips pinch the bowel. **(B)** Shows preparation 2, where the colon has force transducer clips attached to the serosal surface, but no resting tension is applied to the colon. **(C)** Preparation 3, shows the colon tied to an infusion bath with suture thread. **(D)** Preparation 4 shows the oral and anal ends of the colon tied (as in preparation 3), but this preparation is also pierced with hooks and attached to force transducer under 1 gm resting tension.

The empty colon was anchored with a single pin at the oral and anal ends (see Figure 1A, preparation 1), and three 10 min video recordings were made. The colon was then either “stimulated” with conventional clips (usually used to make tension recordings) attached to the external surface of the colon, at the oral and anal ends (See Figure 1B, preparation 2), or by anchoring the colon in a fluid infusion bath with cotton thread (See Figure 1C, preparation 3). Ten minute video recordings were made of the stimulated colon also. A fourth type of “stimulation” was applied in the form of stainless steel micro hooks that were attached through the serosa and musculature of the colon (See Figure 1D, preparation 4), which were connected to force transducers to measure contractions.

### Video imaging of CMMCs and generation of spatio-temporal maps

The contractions of the circular muscle of the gut wall were recorded using the Gastrointestinal Motility Monitoring system (GIMM; Med-Associates Inc., Saint Albans, VT, USA). The colon was illuminated from beneath and a digital video camera was used to record the diameter changes in the colon wall. Spatiotemporal maps of the changes in the circumferential diameter (D-maps) were constructed from the digital videos. This was performed using software from the GIMM which converts the video image to a silhouette enabling the number of pixels (shadow caused by colon) to be calculated at each point along the colon, and then represented in the D-map as a grayscale, displaying which regions of the colon were undergoing a diameter change (in mm).

### Mechanical recordings of circular muscle contractility during CMMCs

We recorded the force generated during each CMMC contraction, using independent isometric recording transducers (Grass FT-03C; Grass, Quincy, MA, USA) connected via fine suture. Each force transducer was connected to two custom made preamplifiers (Biomedical engineering, Flinders University) and then to a Powerlab (model: 4/30; AD Instruments, Bella Vista, NSW, Australia). Labchart version 6.0 (AD Instruments, Australia) was used for acquisition and analysis of data. To record circular muscle contractility 1 gm of resting tension was applied to preparations of colon at three sites, one site in the proximal colon, one in the mid region and one in the distal colon (as represented by Figure 1D). In a second type of preparation, we attached stainless steel spring clips to the bowel wall in the proximal and distal colon, as is represented by Figure 1B. In these preparations, no resting tension was applied to the circular muscle. The aim of this type of preparation was to determine whether the act of simply pinching the colon wall, without any imposed resting tension was sufficient to change CMMC properties.

### Drugs and solutions

The Krebs solution used contained (in mM): NaCl. 118; KCl. 4.7; NaHPO_4_.2H_2_O. 1.0; NaHCO_3_. 25; MgCl.6H_2_O. 1.2; D-Glucose. 11; CaCl_2_.2H_2_O. 2.5. Hexamethonium bromide was purchased from Sigma Chemical Co. St. Louis. MO USA.

### Measurements and statistics

Spatio-temporal D (diameter)-maps generated from video recordings were used to calculate the number of CMMCs over a 10 min period, including the extent of propagation and velocity (mm/s) of each contractions. Velocity was calculated using the scale produced with each map [showing distance (mm) and time(s)] to determine the time taken (s) for the full contraction to take place, and over what distance of the colon (mm) it occurred. Data in the results section are presented as means ± s.e.m. The use of “*N*” in the results section refers to the number of animals on which observations were made. Statistical analysis of results was conducted using Student's paired *t*-tests. *P*-values < 0.05 were taken as being statistically significant.

## Results

### General observations

The entire colon was removed from 24 C57BL/6 control mice. Each preparation was found to have, a mean of 4 discrete fecal pellets within the colonic lumen (range of 1–7, *N* = 24). Once the whole colon, containing pellets had been placed into the organ bath at 35–36°C, it was found that in 5 of these 24 preparations all pellets were naturally expelled within 30 min. Three preparations did not expel any pellets from the lumen and in the remaining preparations, some, but not all pellets were propelled along the length of the large intestine, until expulsion from the lumen occurred. All of the 24 preparations contained at least 1 natural pellet at the time of removal of the preparation from the animal. Irrespective of whether all pellets had been naturally expelled from each preparation, all colon preparations were gently flushed with approximately 5 ml warm Krebs solution. This process was performed in such a way as to not impose any overt distension of the colonic wall.

### Expulsion of natural faecal pellets

**Figure 2:**
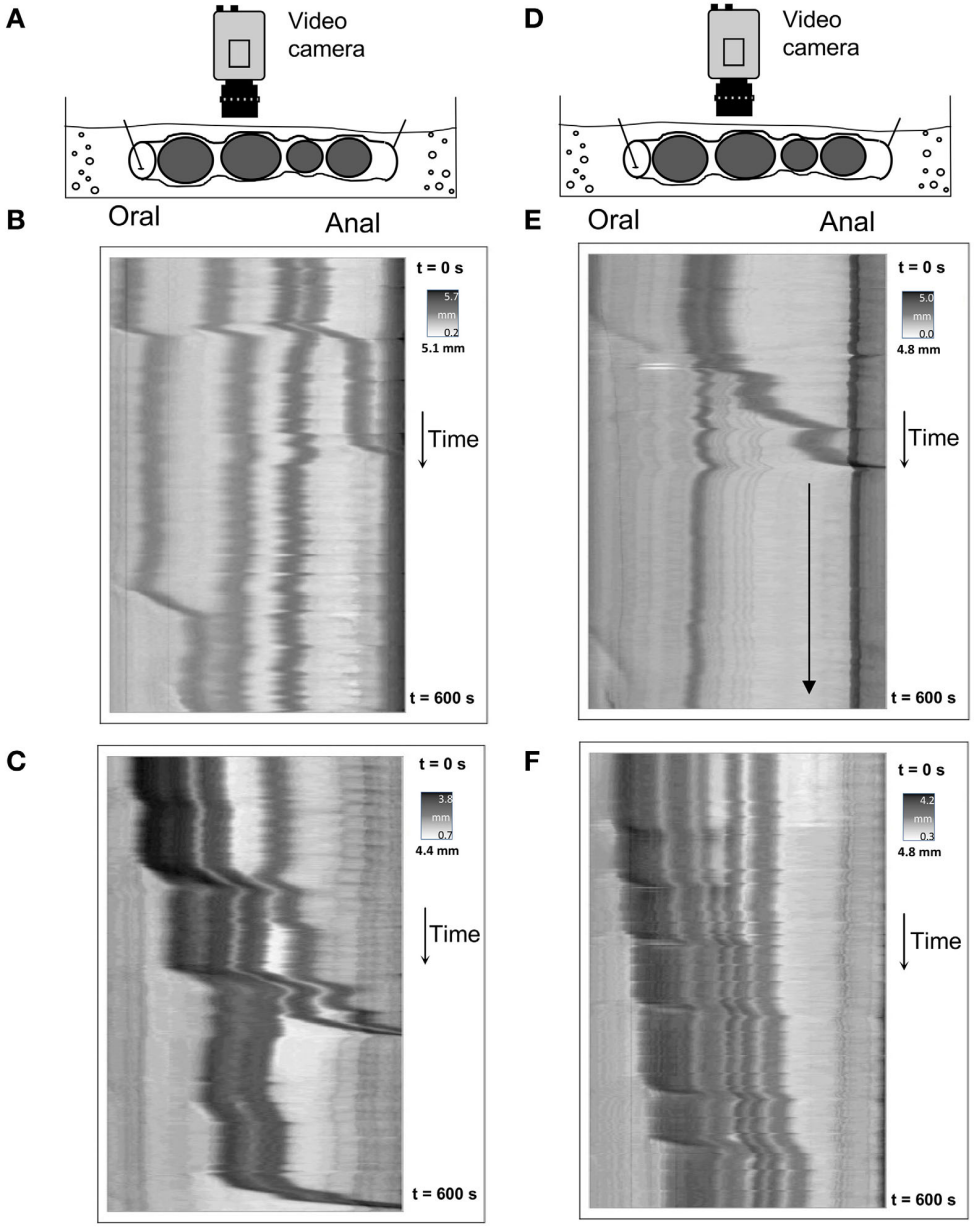
**Spatiotemporal maps of colonic circumferential wall diameter in 4 separate mice, showing propulsion of natural pellets in an aboral direction along the full length of isolated colon. (A,D)** are diagrammatic representations of the preparations of colon that contain endogenous fecal pellets and were used to record changes in circumferential diameter by a video camera mounted directly above the preparation. These were then converted to the D-maps as shown in **(B,C,E,F)**. CMMCs occur which induce pellet movement, with some pellets being propelled significant distances aborally. **(E)** Shows that after a single pellet has been expelled, the colon is empty and becomes relatively silent (indicated from the point at the downward arrow on the map).

Using the preparation described in Figure 1A, video imaging was performed on isolated whole colon preparations that contained multiple natural endogenous fecal pellets. Under these conditions, the whole isolated mouse colon generated CMMCs at an average rate of 1.9 ± 0.1 min^−1^, where each contraction was initiated in the proximal colon and propelled anally, usually the entire distance to the anus (*N* = 24). In most cases, each CMMC contraction propagated over fixed pellets; and did not lead to substantial aboral movement of any single pellet along the colon. Figure 2 shows spatiotemporal maps generated from 4 separate colonic preparations. Figure 2F shows CMMCs that propagate over fixed pellets, but no substantial movement or expulsion of any pellets occurs. It was found that CMMCs traversed an average of 99.5 ± 0.4% of the length of the colon. The velocity of these individual CMMCs was 2.2 ± 0.2 mm/s. Hexamethonium (250 μM) immediately prevented propulsion of all faecal pellets (*N* = 6).

### Characteristics of CMMCs in empty segments of mouse colon

**Figure 3:**
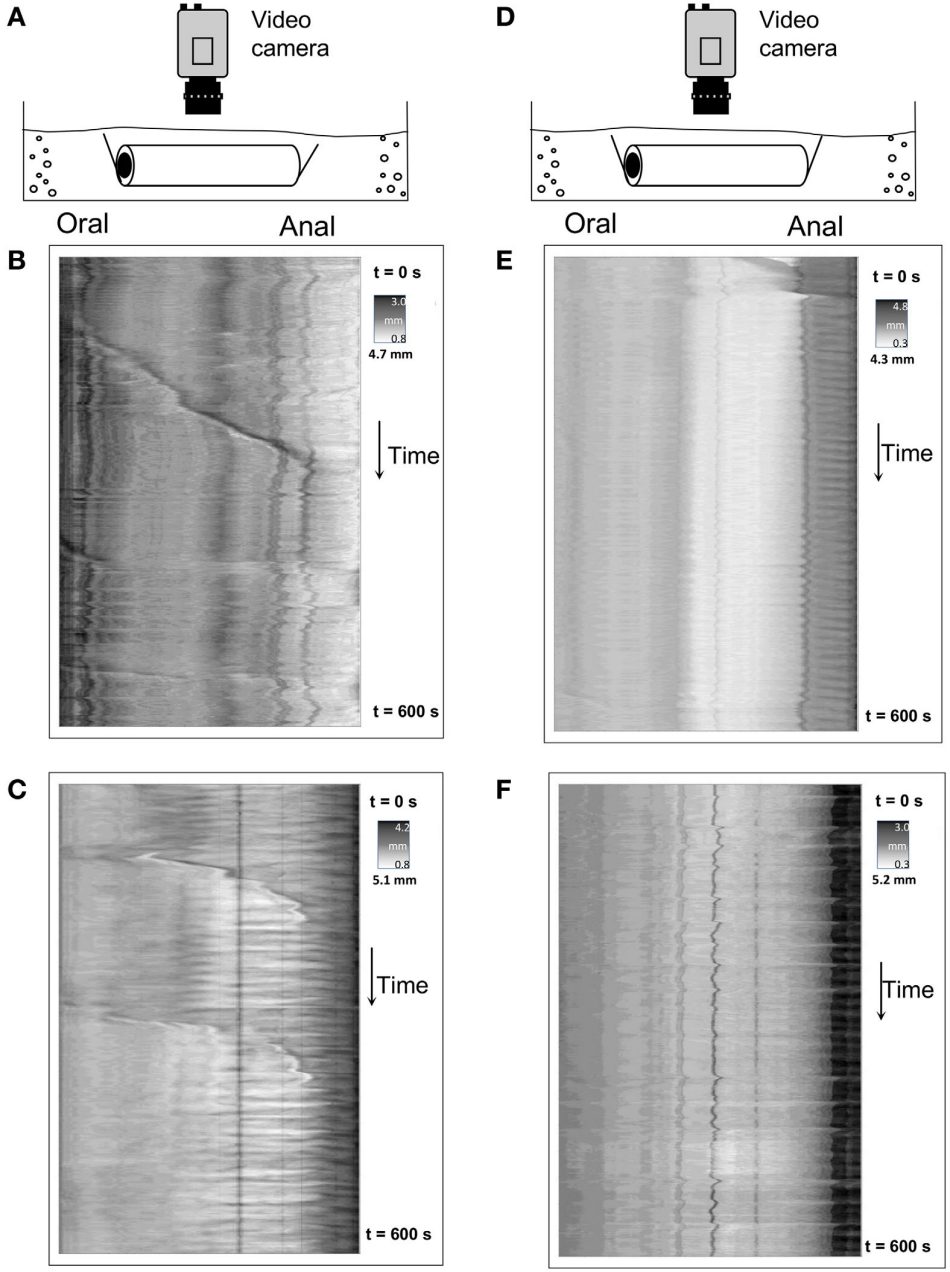
**Spatiotemporal maps of colonic wall diameter showing CMMCs in 4 separate empty colonic preparations. (A,D)** are diagrammatic representations of the empty preparations, with the video camera mounted directly above each preparation. **(B,C)** Show infrequent CMMCs contractions propagating at a slower velocity, that typically do not propagate over the full length of colon. **(E)** Shows the absence of contractions in an empty colon. **(F)** has no obvious CMMCs present.

In the same preparations that demonstrated consistent CMMCs when multiple fecal pellets were present, we were particularly interested in whether CMMCs would still occur when these same preparations were devoid of any fecal content. It was found, that in these empty preparations CMMCs either did not occur, (11/24 preparations) shown in Figures 3B–F, or, occurred at a significantly reduced frequency (mean frequency: 0.18 ± 0.02 min^−1^; *N* = 13) as shown by Figures 3B,C. Also, interestingly, the extent of propagation of individual CMMCs was significantly decreased in empty preparations, to 48.2 ± 9.3% of the length of colon. The velocity of these CMMCs also was significantly slower than in full colonic preparations (mean velocity: 0.7 ± 0.1 mm.s^−1^; *P* < 0.05; *N* = 13). To test whether CMMCs in an empty intestine were of neurogenic origin, we again applied hexamethonium to the organ bath. In 4/4 empty preparations HEX (250 μM) abolished all propulsive CMMCs under these conditions.

### Impact of conventional recording techniques on CMMC frequency and their characteristics of propagation

**Figure 4:**
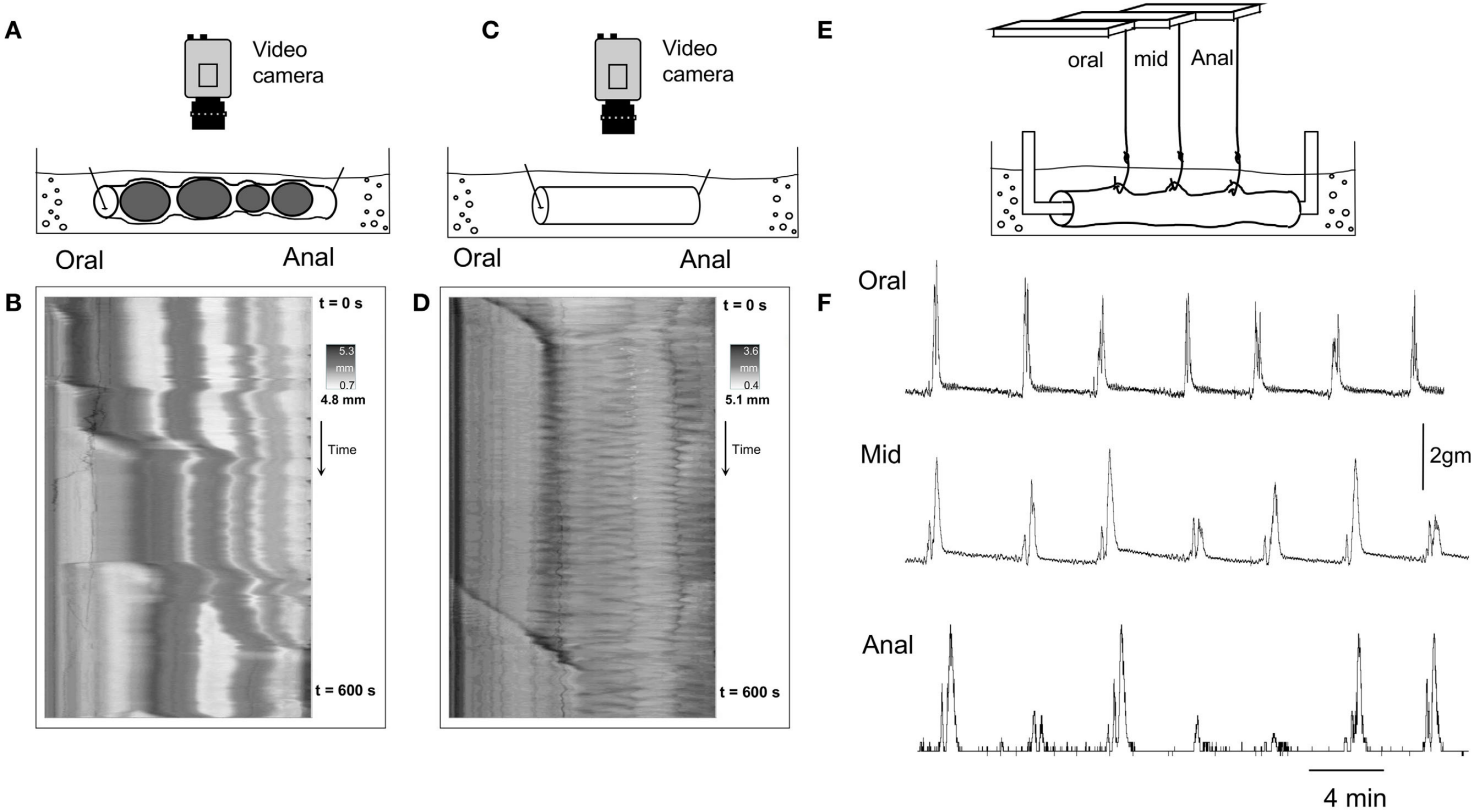
**(B,D,F)** are three separate recordings, from the same segment of whole mouse colon, using different recording methodologies. **(A)** Shows a diagrammatic representation of the preparation from which video recordings were made from a whole colon that contained multiple fecal pellets. **(B)** Shows the D-map from the same preparation, with CMMCs present that propel a number of pellets aborally. **(C)** Shows the preparation from which video recordings were made from the same segment of colon, but devoid of all fecal pellets. **(D)** Shows a D-map from the same preparation of colon as in **(B)** but the colon is now devoid of all fecal pellets. CMMCs are considerably less frequent. **(E)** Shows a diagram of the preparation but now when isometric force transducers are attached to the circular muscle at three sites along the colon, with 1 gm resting tension imposed. **(F)** Shows that under these recording conditions, CMMCs are now regularly recorded which propagated from oral, to mid, to distal colon.

**Figure 5:**
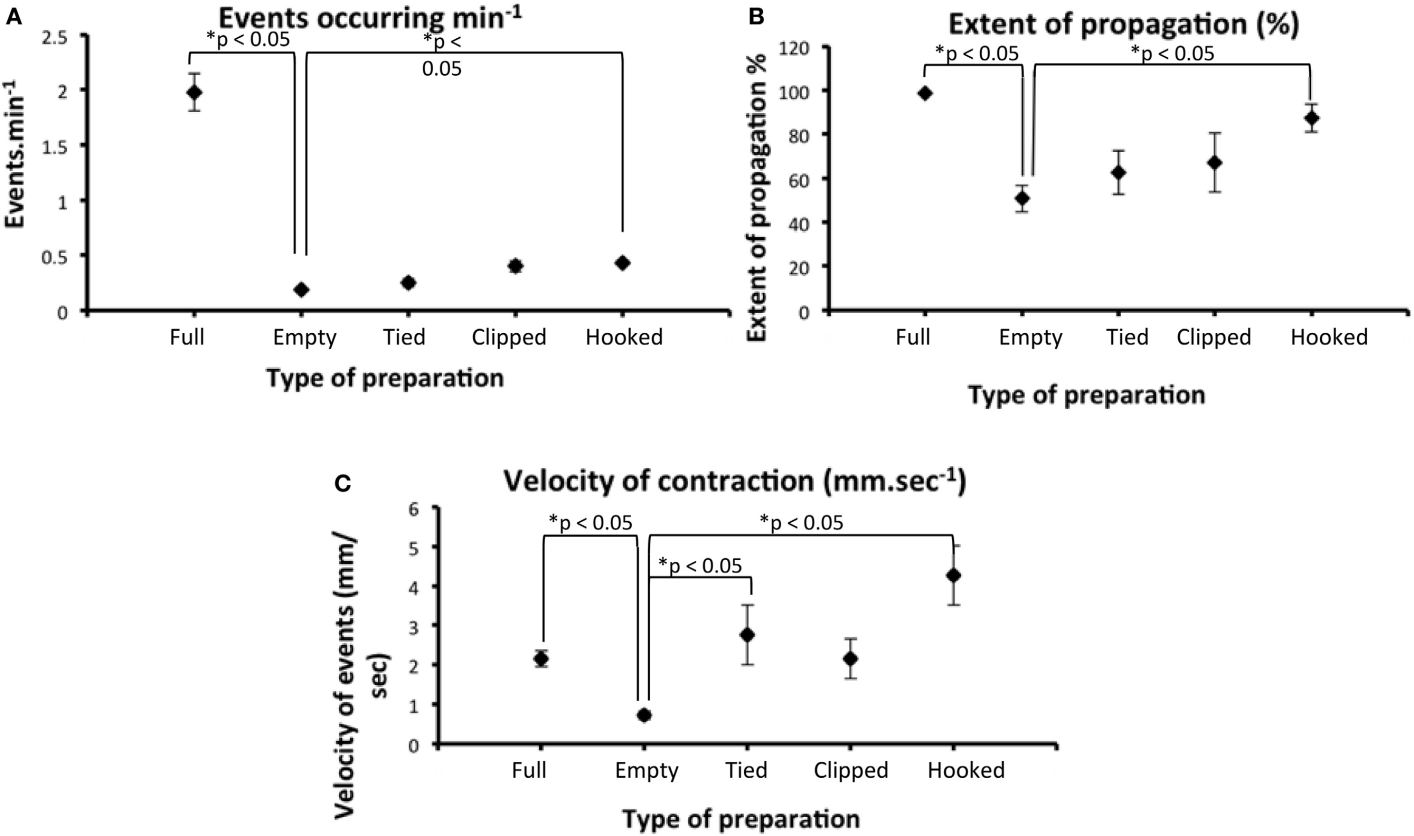
**There was a statistically significant reduction in the number of CMMCs that occurred per minute in full segments of colon (that lacked luminal contents) compared with the same segments that contained multiple endogenous pellets. (A)** empty preparations which had hooks applied and 1 gm tension imposed induced a significant increase in CMMC frequency (compare Empty data set with Hooked data). “Tied” refers to when the colon is cannulated at the oral and anal ends with suture thread and “clipped” refers to when the preparations have stainless steel clips to pinch the bowel wall (e.g., Figure 1B). **(B)** the extent of propagation of individual CMMCs was significantly increased preparations were hooked and resting tension imposed, compared with the same preparations empty. **(C)** There was a statistically significant reduction in the propagation velocity of CMMCs between full and empty preparations. When these same preparations were then stimulated with one of the two conventional techniques, tied or hooked, CMMC velocity significantly increased.

Since we found that CMMCs occurred either significantly less frequently, or not at all, in an empty segment of colon, we then investigated whether conventional organ bath recording techniques, including isometric force transducers and an imposed degree of resting tension on the circular muscle, may induce the CMMC motor pattern. To do this, we used different preparations 2, 3, and 4 in Figure 1. Firstly, when empty preparations were stimulated by attaching tension recording clips at two sites along the colon (as in Figure 1B), the frequency of CMMCs, their extent of propagation, and the velocity of propagation did not change (*N* = 5). In a separate cohort of experiments, we cannulated the oral and anal ends of the empty segments of colon after mounted into an organ bath (see Figure 1C). In these experiments, it was found that the frequency of CMMCs remained similar, as did the extent of propagation, however the velocity of CMMCs significantly increased to 2.75 ± 0.7 mm.s^−1^ (See Figure 5). Another cohort of empty preparations were then studied where the preparations were mounted into the organ bath with oral and anal ends cannulated with suture thread, and then metal hooks attached to the colon for mechanical recordings where 1 gm resting tension was applied (as in Figure 1D). In these experiments, the frequency of CMMCs significantly increased to (0.43 ± 0.025 min^−1^), as did their extent of propagation (87.3 ± 6.3%) and propagation velocity (4.3 ± 0.7 mm.s^−1^) in comparison to empty preparations. This implies that the tension and resulting stretching of the gut applied during this recording procedure is the key factor in causing the change in CMMC characteristics. This notion is best exemplified in Figure 4, where CMMCs are observed expelling natural pellets (Figures 4A,B), then the relatively quiescent behavior is observed in the same preparation in the empty state (Figures 4C,D). Finally, a pronounced increase in CMMC frequency occurs (Figure 4F) when resting tension is increased by the hooks used to make mechanical recordings (Figure 4E). In 11/11 preparations where CMMCs were evoked by any of the conventional techniques described above using clips or hooks, all CMMC activity was abolished in the presence of HEX (250 μM).

## Discussion

The major finding of the current study was the unexpected observation that CMMCs occur infrequently, or not at all, in empty segments of whole isolated mouse colon, provided that conventional recording methods were not used. Also, this study revealed that in empty preparations of colon, CMMCs were found to propagate over short distances of bowel and at significantly reduced velocities. This is a major observation which is in direct contrast to the current belief that CMMCs occur frequently in empty segments of isolated whole mouse large intestine, and that they do not require any form of stimulation for their generation. It is important to reconcile the methodological differences between this study and those used in previous studies and why these differences lead to such pronounced changes in CMMC characteristics.

### Comparison of current findings with previous studies

Many studies have consistently demonstrated that CMMCs can be regularly recorded from isolated segments of whole mouse colon that lack any fecal content (Fida et al., 1997; Brierley et al., 2001; Powell and Bywater, 2001; Spencer and Bywater, 2002; Spencer et al., 2005; Roberts et al., 2008; Spencer et al., 2013). These studies have repeatedly shown that CMMCs occur in empty segments of colon, occurring approximately every 1–3 min; and are dependent upon the enteric nervous system for their generation (Fida et al., 1997; Brierley et al., 2001; Spencer and Bywater, 2002; Powell and Bywater, 2003; Roberts et al., 2008) The presumption has been therefore that the presence of luminal content (fecal pellets) is not required for CMMC generation. Whilst there has been no doubt that these previous studies lacked luminal contents, there is also no doubt that some form of stimuli has been applied to the colon to enable recordings of CMMC activity. This has usually been in the form of isometric force transducers that imposed a degree of stretch on the colon and mechanical clips that pinch the intestine, or, simply by mounting the oral and anal ends of the intestine into the organ bath to infuse intraluminal contents. The major difference between the current study and all previous reports of CMMC activity is that in this study we did not use clips to pinch the bowel, nor impose any resting tension on the musculature, nor mount the oral and anal ends of the colon in a conventional recording system for infusing intraluminal fluid. Instead of mounting the colon into an organ bath using conventional anchor points at the oral and anal ends, we used a single pin to anchor the oral and anal ends of the colon to the base of the sylgard lined organ bath. This minimized external stimulation of the preparation.

### Change in CMMC frequencies between preparations that contain multiple fecal pellets and preparations free of luminal contents

In this study, we observed that in whole colons that contained multiple fecal pellets, CMMCs occurred frequently, with a mean interval of ~30 s (1.9 ± 0.1 CMMC.min^−1^). However, when the same segments of colon were flushed free of all luminal content, CMMCs either did not occur, or, if they were present, occurred significantly less frequently (mean: 0.16 ± 0.02 min^−1^). It would be a reasonable assumption that the frequency of occurrence of CMMCs would be higher when content is present in the lumen to facilitate the evacuation of content from the colon, and conversely lower in frequency, or absent, when the colon is free of content. In hindsight, it is now clear to us that the reason why CMMCs occurred in our previous studies at relatively high frequencies (~1–3 min), is because the colonic preparations had been stimulated by the methodology used to record CMMCs from these segments of bowel see: Fida et al. (1997); Powell and Bywater (2001); Spencer and Bywater (2002); Spencer et al. (2013). It is known that the mechanisms by which stretch on the luminal wall increases CMMC frequency is not dependent upon the mucosa or submucosal plexus because circumferential stretch still increases CMMC frequency when the mucosa and submucosal plexus is removed (Zagorodnyuk and Spencer, 2011).

### Differences in the extent of propagation of CMMCs in empty vs. full segments of colon

We were particularly interested in whether the extent of propagation of CMMCs in isolated full length preparations that contained endogenous fecal pellets would show similar characteristics as CMMCs recorded from empty segments of colon. It was found that in whole colons that contained endogenous pellets, CMMCs propagated along the full length of colon. In contrast, in the same preparations of colon that had expelled all content, the extent of propagation of CMMCs decreased to 48% of their original length. In only 1 of 24 empty preparations was a single CMMC recorded that propagated the full length along the colon (proximal to mid to distal). This is in contrast with our previous findings that showed CMMCs propagate along the full length of colon in empty segments of colon, when mechanical clips were used to pinch the bowel enabling tension recordings to be made. Taken together, the presence of spring clips, or hooks that pinch or stimulate the bowel and the imposed resting tension leads to substantial increases in the extent of propagation of CMMCs along the colon. A previous study by Roberts and colleagues demonstrated that cannulating the mouse colon in an organ bath did not significantly alter CMMC frequency (Roberts et al., 2007). This is consistent with our study. We found that only when the isometric force transducers were also attached to the colon and 1 gm resting tension applied was there a significant increase in CMMC frequency (See Figure 5A).

## Conclusion

The findings of this study reveal that in the isolated whole mouse colon, CMMCs occur rarely, or not at all, when the colon is devoid of all natural fecal pellets and provided it is not stimulated by conventional recording methods. This study also shows that the frequency, propagation velocity and extent of propagation of CMMCs along the colon is significantly increased when stretch is applied to the colon, by multiple fecal pellets. We suggest that CMMC frequency, velocity and extent of propagation increase when contents are present in the lumen to facilitate evacuation from the large intestine.

### Conflict of interest statement

The authors declare that the research was conducted in the absence of any commercial or financial relationships that could be construed as a potential conflict of interest.
